# Supplementary material for: Corticosterone Induces HMGB1 Release in Primary Cultured Rat Cortical Astrocytes: Involvement of Pannexin-1 and P2X7 Receptor-Dependent Mechanisms
Source: Cells. 2020 Apr 25;9(5):1068. doi: 10.3390/cells9051068 (PMC7290518; doi:10.3390/cells9051068)
Supplement: Supplementary file 1 [file cells-09-01068-s001.pdf]

Supplemental Fig 1

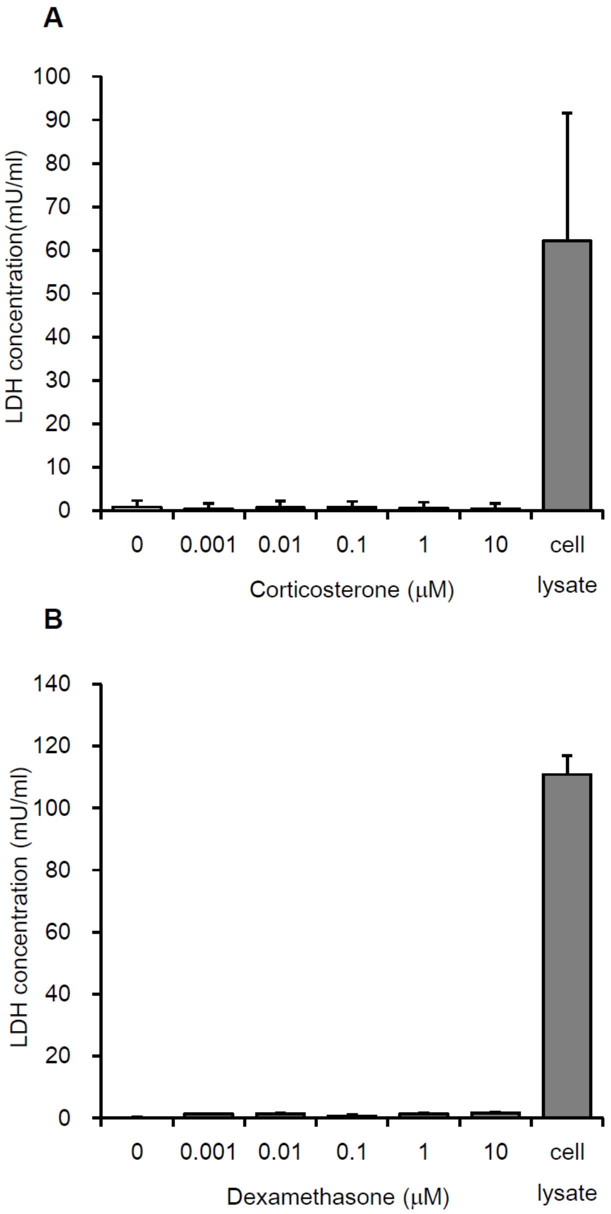

Supplemental Fig. 1. Effect of corticosterone or dexamethasone treatment on LDH release in primary cultured cortical astrocytes. **A**, Astrocytes were treated with the indicated concentrations of corticosterone over a period of 24 h, and LDH release into the media was assessed with the LDH assay. The data are expressed as the mean ±S.E.M. (n=3). **B**, Astrocytes were treated with the indicated concentrations of dexamethasone over a period of 24 h, and media concentrations of LDH was assessed with the LDH assay. The data are expressed as the mean ±S.E.M. (n=3).

Supplemental Fig 2

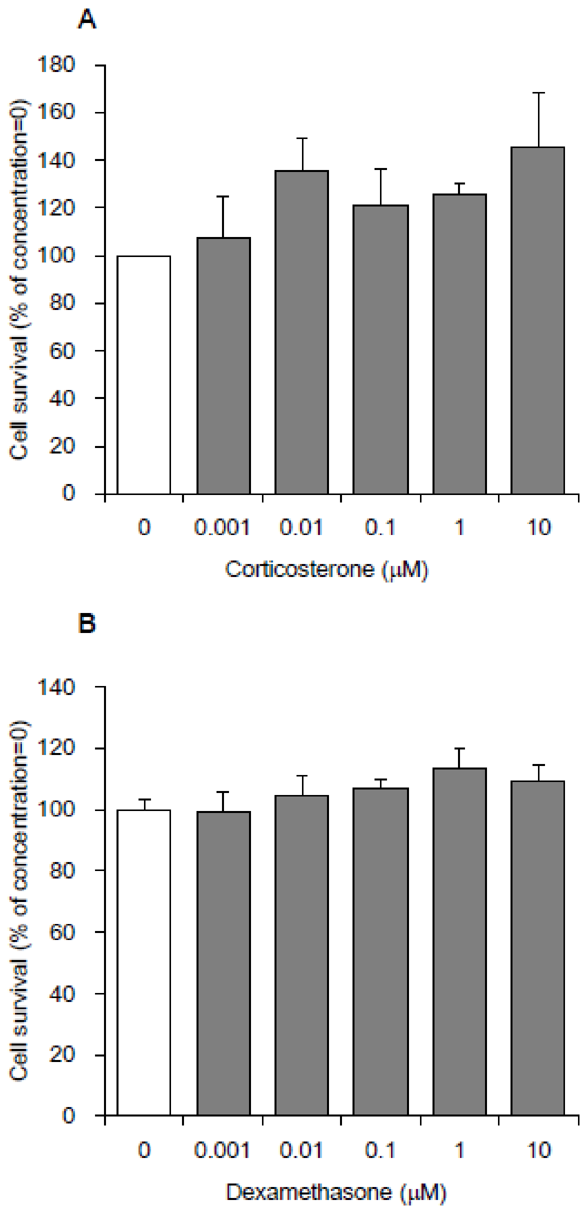

Supplemental Fig. 2. Effect of corticosterone or dexamethasone treatment on cell survival in primary cultured cortical astrocytes. **A**, Astrocytes were treated with the indicated concentrations of corticosterone over a period of 24 h, and cell survival was assessed with the MTT assay. The data are expressed as the mean ± S.E.M. (n=3). **B**, Astrocytes were treated with the indicated concentrations of dexamethasone over a period of 24 h, and cell survival was assessed with the MTT assay. The data are expressed as the mean ± S.E.M. (n=3).
